# Honokiol induces apoptosis and autophagy via the ROS/ERK1/2 signaling pathway in human osteosarcoma cells in vitro and in vivo

**DOI:** 10.1038/s41419-017-0166-5

**Published:** 2018-02-06

**Authors:** Kangmao Huang, Yanyan Chen, Rui Zhang, Yizheng Wu, Yan Ma, Xiangqian Fang, Shuying Shen

**Affiliations:** 1 Department of Orthopaedic Surgery, Sir Run Run Shaw Hospital, Medical College of Zhejiang University, 3 East Qingchun Road, Hangzhou, 310016 China; 2 Department of Surgical Oncology, First Affiliated Hospital, Medical College of Zhejiang University, Hangzhou, 310003 China; 3 Department of Neurosurgery, Children’s hospital of Nanjing Medical University, Nanjing City, China

## Abstract

Osteosarcoma is the most common primary malignant tumor of bone, the long-term survival of which has stagnated in the past several decades. In the present study, we investigated the anticancer effect of honokiol (HNK), an active component isolated and purified from the magnolia officinalis on human osteosarcoma cells. Our results showed that honokiol caused dose-dependent and time-dependent cell death in human osteosarcoma cells. The types of cell death induced by honokiol were primarily autophagy and apoptosis. Furthermore, honokiol induced G0/G1 phase arrest, elevated the levels of glucose-regulated protein (GRP)−78, an endoplasmic reticular stress (ERS)-associated protein, and increased the production of intracellular reactive oxygen species (ROS). In contrast, reducing production of intracellular ROS using *N*-acetylcysteine, a scavenger of ROS, concurrently suppressed honokiol-induced cellular apoptosis, autophagy, and cell cycle arrest. Consequently, honokiol stimulated phosphorylation of extracellular signal-regulated kinase (ERK)1/2. Furthermore, pretreatment of osteosarcoma cells with PD98059, an inhibitor of ERK1/2, inhibited honokiol-induced apoptosis and autophagy. Finally, honokiol suppressed tumor growth in the mouse xenograft model. Taken together, our results revealed that honokiol caused G0/G1 phase arrest, induced apoptosis, and autophagy via the ROS/ERK1/2 signaling pathway in human osteosarcoma cells. Honokiol is therefore a promising candidate for development of antitumor drugs targeting osteosarcoma.

## Introduction

Osteosarcoma, comprising around 60% of all bone cancer, is a high-graded form of primary bone cancer and has the most prevalence^1,2^. In 1970s, surgery alone without further treatments cured less than 20% of patients with osteosarcoma owing to distant metastasis. Metastasis at the time of diagnosis have been found in ~20% patients with such sarcoma, among which the lung is the predominant site of distant disease. In recent years, the advanced radical treatment and chemotherapy applied to osteosarcoma have improved 5-year survival rate to around 70% for localized tumors, but the prognosis for those with unresectable or metastatic cancer remains unsatisfactory^1,2^.

Honokiol (HNK), a biphenolic compound extracted from Magnolia tree, has an extensive application in traditional Chinese and Japanese medicine for thrombotic stroke, the treatment of anxiety, and gastrointestinal symptoms. It is also used clinically owing to its cardioprotective^3,4^, anti-microbial^5^, anti-inflammatory^6,7^, and antiangiogenic properties^8,9^. Recently, its anti-neoplastic properties in vitro against cancer are gradually demonstrated^8–18^. Furthermore, some reported that honokiol has certain effects in vivo in angiosarcoma^8^, colorectal carcinoma^14^, breast cancer^17^, and gastric cancer^18^ as well as restrained bone metastasis in a murine prostate cancer model^16^. Therapeutic effects are greatly improved with the combination of honokiol and various other chemotherapeutic drugs in vitro^9,11,16^ and in vivo^16^. Moreover, the ability to overcome chemoresistance in human multiple myeloma cells has been demonstrated^9^. It is suggested that through activation of distinct apoptotic mechanisms, the cytotoxicity of honokiol is brought about predominantly^8,9,13,14,16^. Its tolerance^8,14,16^, bioavailability in vivo^14^ as well as practicability^19,20^ make honokiol possible to be an attractive novel agent in the treatment for patients with osteosarcoma.

Autophagy is a preserved progression that delivers cytoplasmic contents to lysosomes for degradation through double-membrane vesicles^21^. Autophagic cells can consequently undergo cell survival or death^22^. In particular, autophagy has been found to closely related to apoptosis, which is a kind of programmed cell death. Therefore, in eukaryotic cells autophagy maintains homeostasis between cell survival and death. Microtubule-associated protein 1A/1B-light chain 3 (LC3) is omnipresent distributed in mammalian cells^23^. During autophagy, by recruiting to autophagosomal membranes, a LC3–phosphatidylethanolamine conjugate (LC3-II) is formed through the conjugation between cytosolic form of LC3 (LC3-I) and phosphatidylethanolamine. Thus, autophagy and autophagy-related processes can be monitored by immunodetecting LC3B-I/II. As for mechanisms, mammalian target of rapamycin (mTOR), a serine/ threonine kinase, is considered to be a major negative regulator of cellular autophagy^24^. mTOR signaling is deregulated in many human diseases because of its participating in regulating cell proliferation and growth. Various protein kinases take part in regulating the activation of mTOR^25^. Phosphorylation of mTOR can subsequently be stimulated by the phosphoinositide-3 kinase (PI3K)-mediated activation of protein kinase B (Akt)^25,26^. In contrast, extracellular signal-regulated kinase 1/2 (ERK1/2) signaling adjusts mTOR phosphorylation negatively^27^. Except for apoptosis, autophagy and autophagy-induced cell death are another two important factors when examining cancer drugs.

Reactive oxygen species (ROS), which is the active forms of oxygen, are by-products from cellular metabolism activities^28^. Cell proliferation and differentiation can be promoted by a moderate increase in ROS, while excessive amounts of ROS are able to interfere cellular signaling pathways owing to its oxidative damage to proteins, lipids and DNA^29–31^. Interestingly, accumulating evidence suggests that cancer cells are under increased oxidative stress, which indicates that they are more likely to be destroyed with further ROS existing induced by exogenous agents^32^. Furthermore, ROS can affect varieties of signaling pathways including MAPK signal transduction cascades^33,34^. As a member of the MAPK family, ERK is of critical importance in various cellular events, such as apoptosis and autophagy^35,36^. Accordingly, it might be beneficial in the treatment of cancer with the good use of targeted inhibition of related signaling pathways, particularly the ROS/ ERK signaling.

In this study, we attempted to evaluate the inhibitory effects of honokiol on osteosarcoma cell lines and primary cells in vitro and in vivo. We further explored the molecular mechanisms, that is, induction of G0/G1 phase arrest, apoptosis and autophagy mediated by the ROS/ERK signaling pathway.

## Results

### HNK inhibits the proliferation of osteosarcoma and less cytotoxic to fibroblasts

**Fig. 1:**
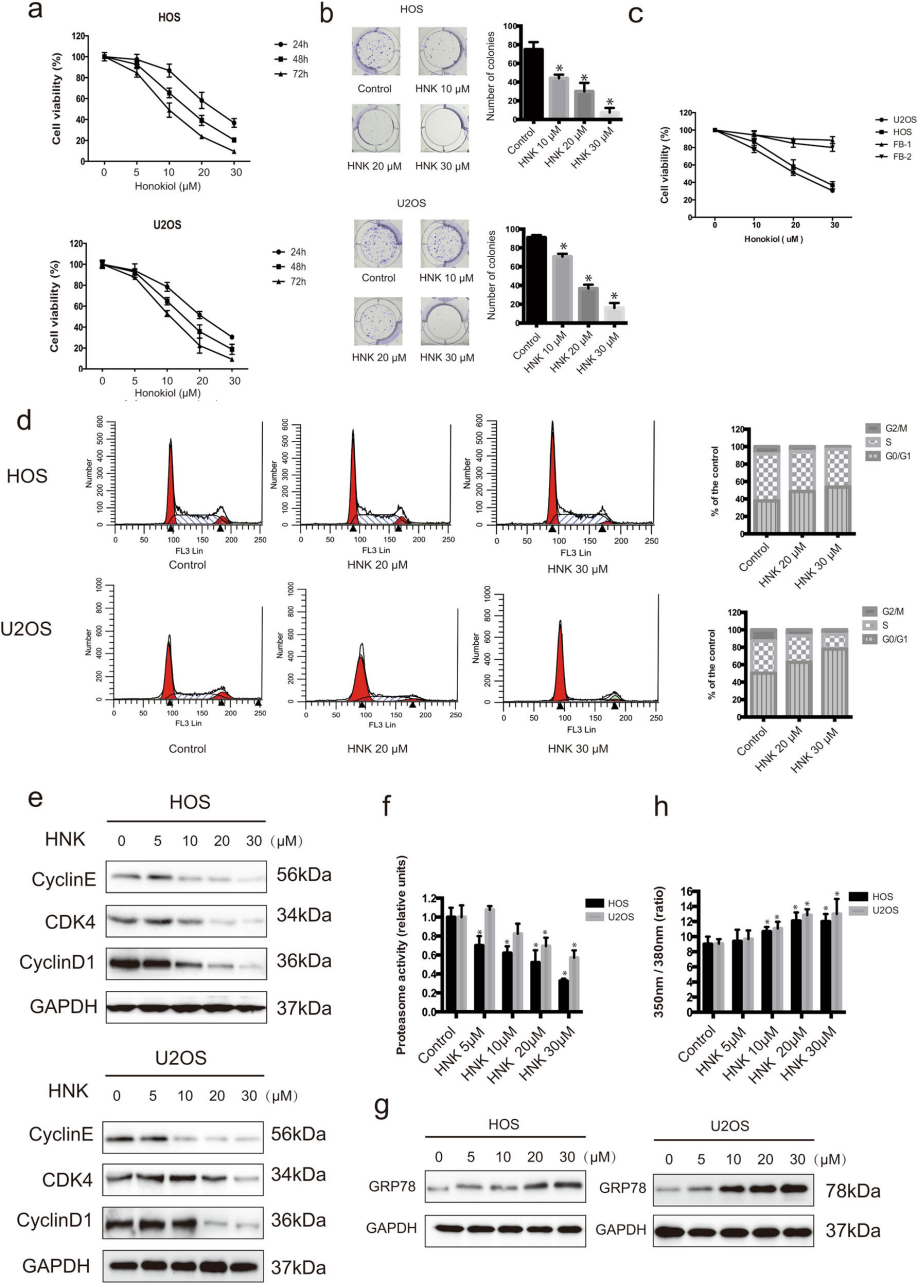
Cytotoxic effects, G0/G1 phase arrest, proteasome activity and ER stress resulting from HNK treatment in osteosarcoma cells **a** The anti-proliferative effect of HNK on osteosarcoma cell lines was determined by MTT. Cells were treated with various concentrations of HNK for 24, 48, and 72 h. Control group contained 0.1% DMSO. Data represented the mean of five replicates. **b** Colony-formation assay of HOS and U2OS cells with control or HNK. **c** Comparison of the effect of HNK on two normal human primary skin fibroblast samples with that on osteosarcoma cells for 24 h. **d** HNK-induced G0/G1 phase arrest. Cells were treated with control or HNK for 24 h and analyzed by flow cytometry. **e** HOS and U2OS cells were treated with HNK for 24 h. The expressions of cell cycle-regulated proteins were measured by western blot. **f** Intracellular proteasome activity in HOS and U2OS cells after treatment with HNK. Cells were treated with 5, 10, 20 or 30 μM HNK for 24 h. *p < 0.05, HNK vs. control. **g** Western blot for GRP78 in HOS and U2OS cells after treatment with HNK. Cells were treated with 5, 10, 20 or 30 μM HNK for 24 h. **h** The intracellular Ca^2+^ concentration in HOS and U2OS after treatment with HNK. Cells were treated with 5, 10, 20 or 30 μM HNK for 24 h. The ratio of 350 to 380 nm was measured by an ELISA reader. *p < 0.05, HNK vs. control. Data are presented as the mean ± s.d. of three independent experiments. Semi-quantification of western blot bands is presented in Figure S1a–d

To assess the effect of HNK on growth of osteosarcoma, HOS and U2OS cells were exposed to various concentrations *of HNK* for 24, 48 or 72 h (Fig. 1a). The IC_50_ values of HNK for 24 h were 17.7 μM for HOS and 21.5 μM for U2OS cells. Colony-formation assay showed fewer colonies formed after HNK treatment (Fig. 1b). Interestingly, human fibroblasts showed strong resistance to HNK, the IC_50_ values for which were 118.9 and 71.5 μM, respectively (Fig. 1c). These results indicate that HNK inhibits the proliferation of osteosarcoma cells (HOS and U2OS) in a dose-and time-dependent manner. Besides, HNK showed less cytotoxic against fibroblasts when compared with osteosarcoma cells in a dose-dependent manner.

### HNK induces G0/G1 phase arrest by regulating cell cycle-regulated proteins

To determine whether HNK inhibits cell proliferation by inducing cell cycle arrest, we examined the distribution of cell cycle in HOS and U2OS cells treated with HNK. As shown in Fig. 1d, HNK led to the accumulation of cells in G0/G1 phase and a corresponding decrease in G2/M and S phases in both HOS and U2OS cells. To elucidate the mechanisms, we measured the expressions of cell cycle-regulated proteins. HNK downregulated the levels of Cyclin E, CDK4 and Cyclin D1 (Fig. 1e). All these results indicate that HNK induces G0/G1 phase arrest by altering the key molecules of G0/G1 cell cycle regulator markers.

### Proteasome activity in osteosarcoma cells treated with HNK

We then detected the proteasome activity in osteosarcoma cells treated with HNK. Figure 1f shows the proteasome activity of cells under the treatment of HNK. Proteasome inhibition in HOS cells occurred at 5, 10, 20 and 30 μM of HNK. Furthermore, we found that the level of proteasome inhibition in HOS cells was higher than in U2OS cells. Previous studies have demonstrated that proteasome inhibition induces cell death through ER stress^37,38^. Therefore, to detect the expression of the ER stress-related protein GRP78, we performed western blot with lysates from cells receiving each of the different concentrations of HNK (Fig. 1g). Our results showed that GRP78 expression increased with HNK treatment compared with control, thus confirming that the HNK-induced ER stress in HOS and U2OS cells. It has been reported that ER stress leads to a release of calcium^39^. To examine whether HNK increases the concentration of intracellular Ca^2+^, we measured the change in intracellular Ca^2+^ with fura 2-AM, a plasma membrane-permeable Ca^2+^-fluorescent probe^40^. HNK increased intracellular Ca^2+^ in both HOS and U2OS cells (Fig. 1h).

### HNK induces mitochondria-mediated apoptosis

**Fig. 2:**
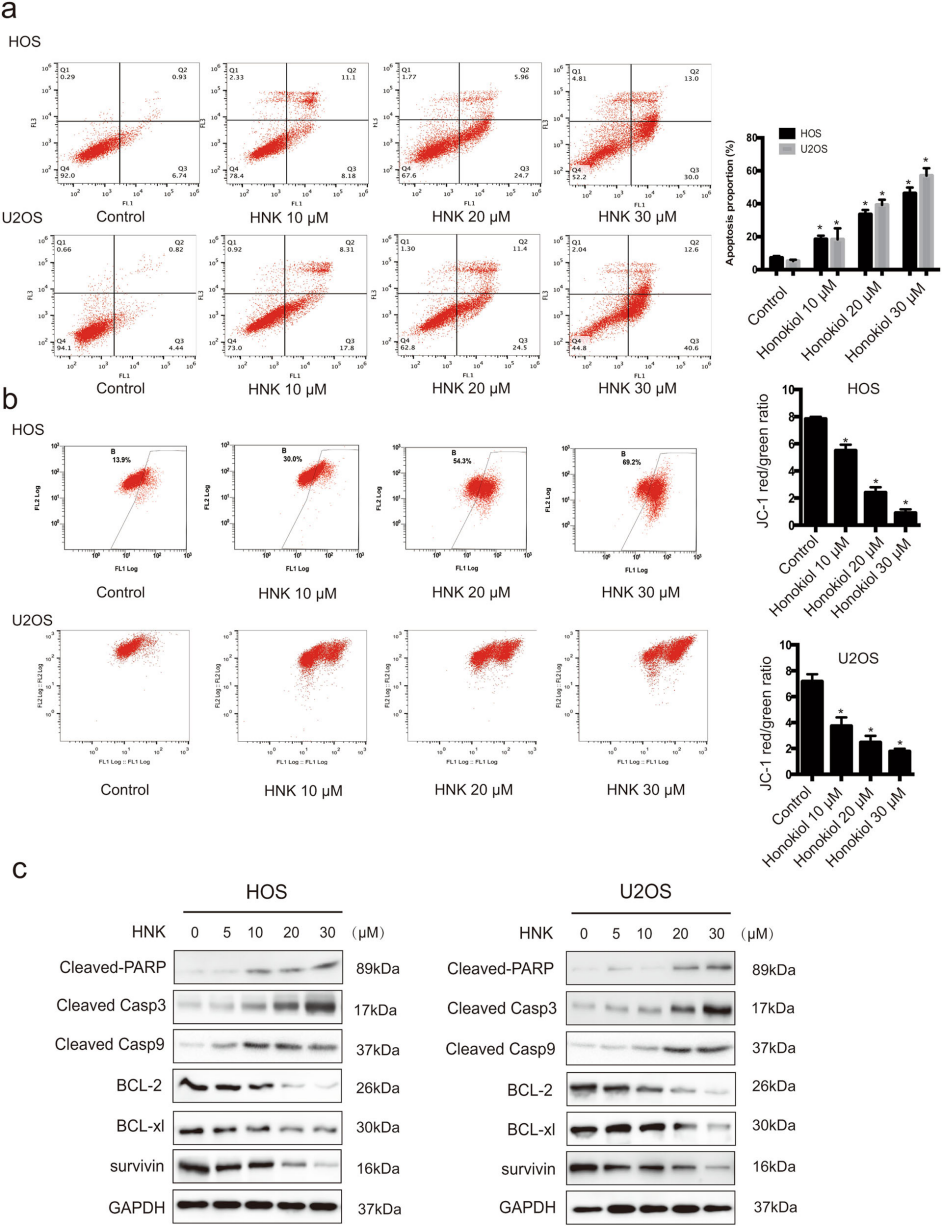
Evidence that HNK induces apoptosis in osteosarcoma cells **a** HOS and U2OS cells treated with HNK were stained with annexin V-FITC/PI and analyzed by flow cytometry. The chart illustrates apoptosis proportion from three separate experiments. **b** The mitochondrial membrane potential was measured with JC-1 fluorescent probe and assessed by flow cytometry. The chart illustrates changes of JC-1 red/green rate from three independent experiments. **c** Cells were treated with various concentrations of HNK for 24 h. The expressions of cleaved PARP, caspase-3, caspase-9, BCL-2, BCL-xl, and survivin were determined by western blot. Semi-quantification of western blot bands is presented in Figure S1e, f

It is well established that during ER stress, cytosolic calcium released from the ER is taken up by the mitochondrion to stimulate ROS overgeneration and the release of cytochrome *c*, both of which lead to apoptosis^39,41^. To determine whether apoptosis is responsible for the inhibition of cell growth induced by HNK, we performed flow cytometry assay. To quantify the apoptosis, cells treated with HNK were stained with annexin V-FITC/PI. Figure 2a indicates that the proportion of apoptosis was negligible for control cells, whereas 24 h of exposure of cells to HNK resulted in a dose-dependent increase of early apoptotic cells. Next, we investigated the effect of HNK on mitochondria. Figure 2b reveals that mitochondrial membrane potential (MMP) sharply decreased following HNK treatment. We then investigated the expression of downstream apoptotic proteins by western blotting. As shown in Fig. 2c, HNK markedly activated caspase-3, caspase-9 and led to PARP cleavage, while the expression of Bcl-2, Bcl-xl, and survivin was found to be decreased. Overall, these results clearly indicate that HNK induces mitochondria-mediated apoptosis.

### HNK triggers autophagy which contributes to HNK-induced cell death

**Fig. 3:**
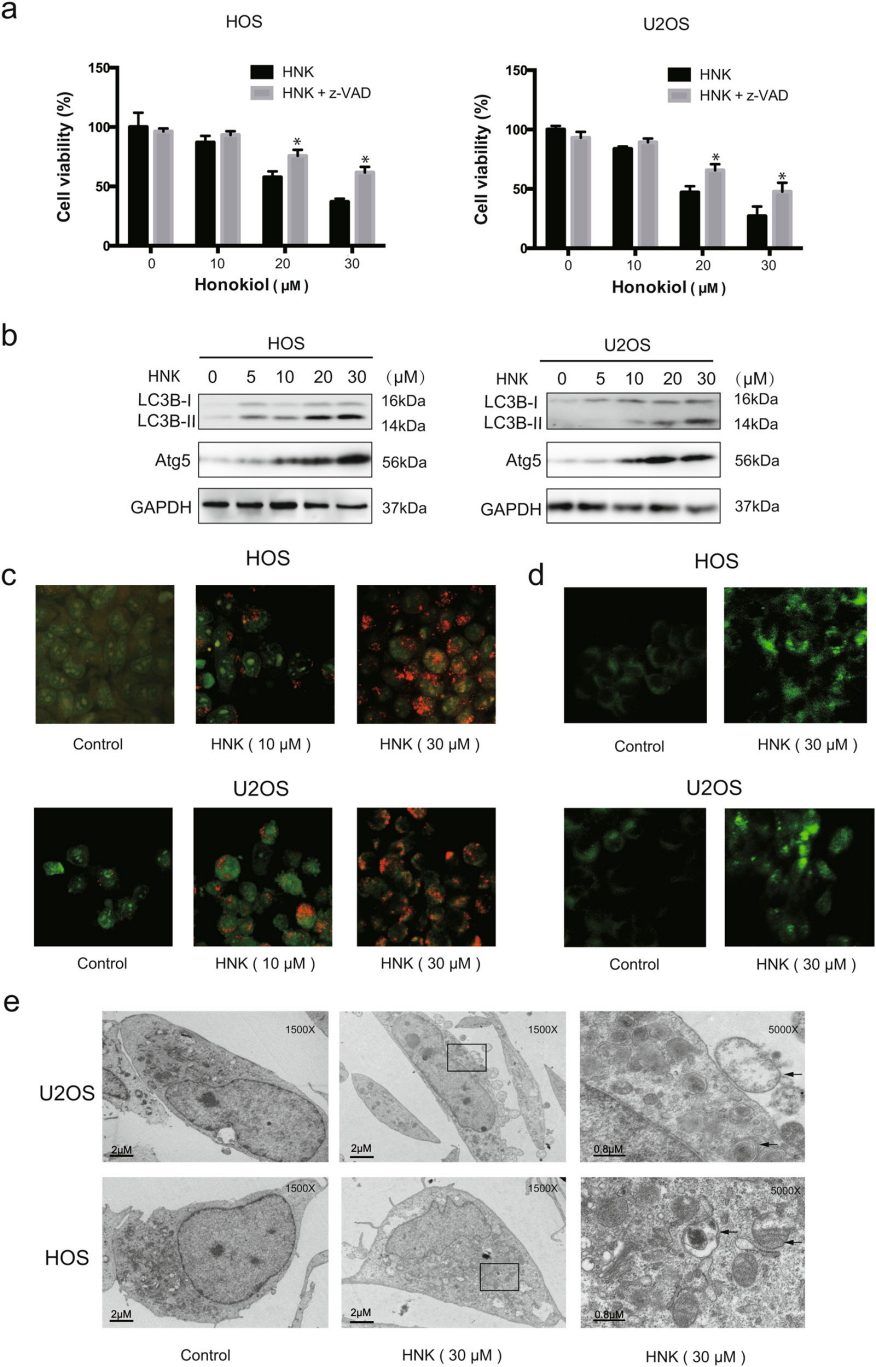
HNK induces autophagy **a** Cells were pretreated with z-VAD-fmk (20 μM) for 2 h and then incubated with control or HNK for 24 h. Cell viability was assessed by MTT. **b** Cells were treated with various concentrations of HNK for 24 h. The level of LC3B and Atg5 was measured by western blot. **c** Cells treated with or without HNK for 24 h were collected and stained with acridine orange. Representative images of acridine orange-stained cells captured by fluorescent microscopy (×400) are shown. **d** Osteosarcoma cells were treated with HNK for 24 h and stained by MDC. **e** Transmission electron microscopy was utilized to observe the formation of autophagosome and ultrastructural change of nucleus. Arrows indicate autophagosomes containing intact and degraded cellular debris. Semi-quantification of western blot bands is presented in Figure S1g, h

To understand the role of apoptosis in the HNK-induced cell death, we examined cell viability in the presence of z-VAD-fmk. Unexpectedly, we found that z-VAD-fmk only caused a partial reduction in the HNK-induced cell death (Fig. 3a), implying that other forms of cell death may be involved. Then we measured the autophagy marker protein LC3B to determine whether autophagic cell death was induced. Figure 3b shows that HNK increased the level of LC3B-II and Atg5 in HOS and U2OS cells. We also observed that HNK led to the accumulation of bright red acidic vesicles resembling autolysosomes (Fig. 3c). Furthermore, we measured the incorporation of MDC in osteosarcoma cells, a marker for mature autophagic vacuoles (AVs) such as autophagolysosomes. HNK treatment significantly increased the level of MDC-stained AVs in both osteosarcoma cell types (Fig. 3d). TEM was used to directly demonstrate autophagosome formation. Figure 3e shows that, concurrent with apoptotic chromatin condensation, numerous large autophagic vacuoles in the cytoplasm were observed, in which the vacuolar contents were degraded, providing evidence for the impact of HNK in the regulation of autophagic formation in osteosarcoma cells.

### HNK induces ERK activation, which is required in HNK-induced apoptosis

**Fig. 4:**
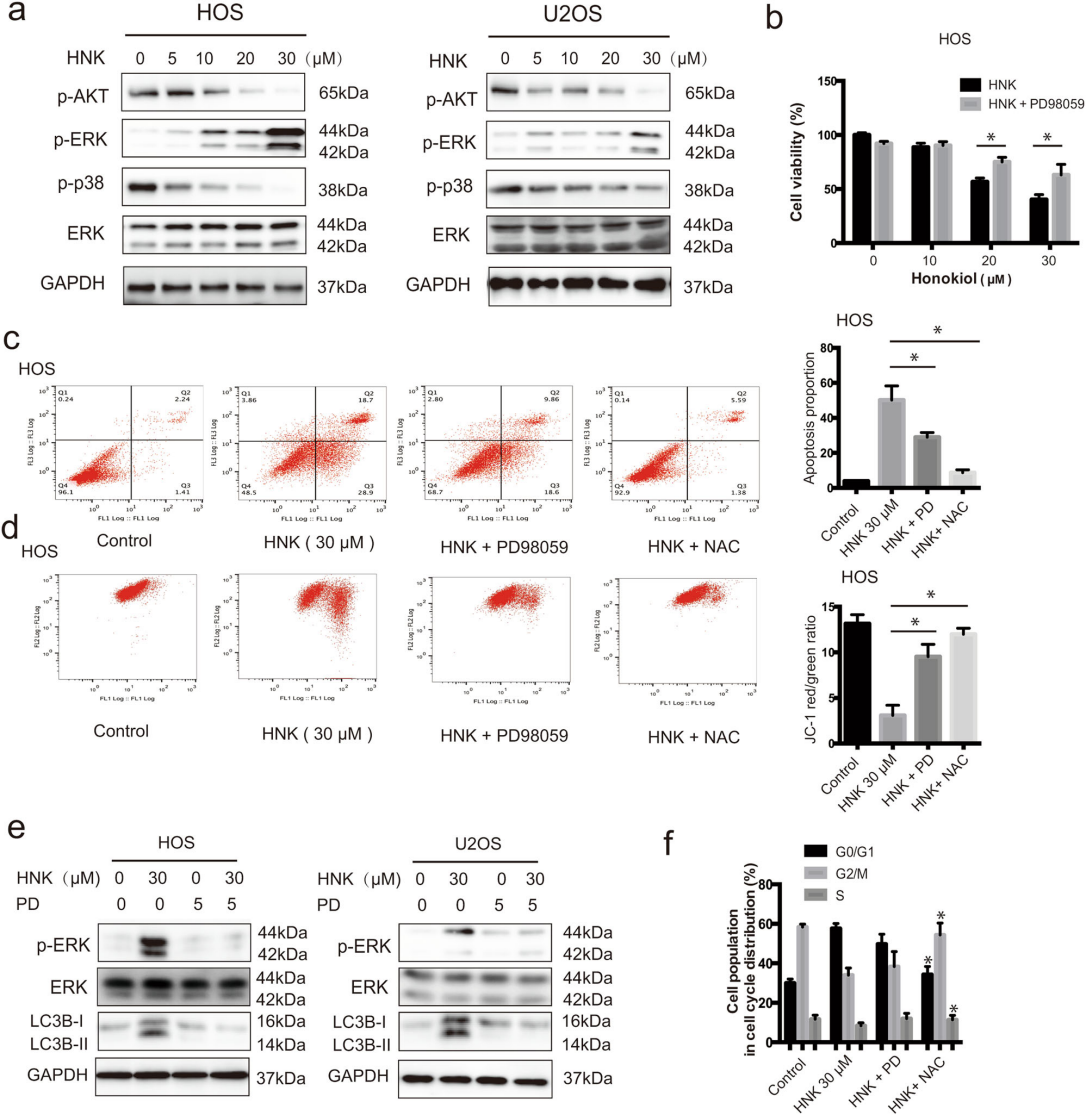
ERK activation are triggered by HNK and roles of ERK in autophagy and apoptosis induced by HNK **a** Cells were treated with various concentrations of HNK for 24 h. Levels of phospho-AKT, phospho-ERK, phospho-p38, and total ERK were determined by western blot. **b** HOS cells were preincubated with PD98059 (40 μM) for 2 h, and then treated with HNK (30 μM) for 24 h. Cell viability was measured by MTT. **c**, **d** HOS cells were preincubated with PD98059 (40 μM) or NAC (5 mM) for 2 h, and then treated with HNK (30 μM) for 24 h. Induction of apoptosis and changes of mitochondrial membrane potential were assessed by flow cytometry. Quantitative analysis in histograms was presented. **e** Cells were preincubated with PD98059 (40 μM) for 2 h, and then treated with HNK (30 μM) for 24 h. Levels of phospho-ERK, ERK and LC3B-II were determined by western blot. **f** Cell cycle was evaluated by flow cytometry. The percentage of cell cycle distribution was presented in histograms. Semi-quantification of western blot bands is presented in Figure S1i, j and S2a, b

We investigated the effect of HNK on ERK activation. Figure 4a shows that HNK increased the level of ERK phosphorylation in both HOS and U2OS cells. To determine the contribution of activated ERK to HNK-induced apoptosis or cell cycle arrest, we used the specific ERK inhibitor, PD98059 (PD). MTT assay showed that PD could effectively reduce the cell death induced by HNK (Fig. 4b). Flow cytometry assay indicated that PD attenuated the HNK-induced apoptosis and inhibited depolarization of mitochondria (Fig. 4c, d). Western blot analysis showed that PD inhibited ERK phosphorylation and activation of autophagy-related proteins to a great extent (Fig. 4e). However, PD failed to restore the HNK-induced increase in the G0/G1 population (Fig. 4f). These results suggest that the activation of ERK is required for HNK-induced apoptosis but not involved in G0/G1 phase arrest.

### ROS is the proximal event of ERK and acts as an initiator in HNK-induced apoptosis and G0/G1 phase arrest

**Fig. 5:**
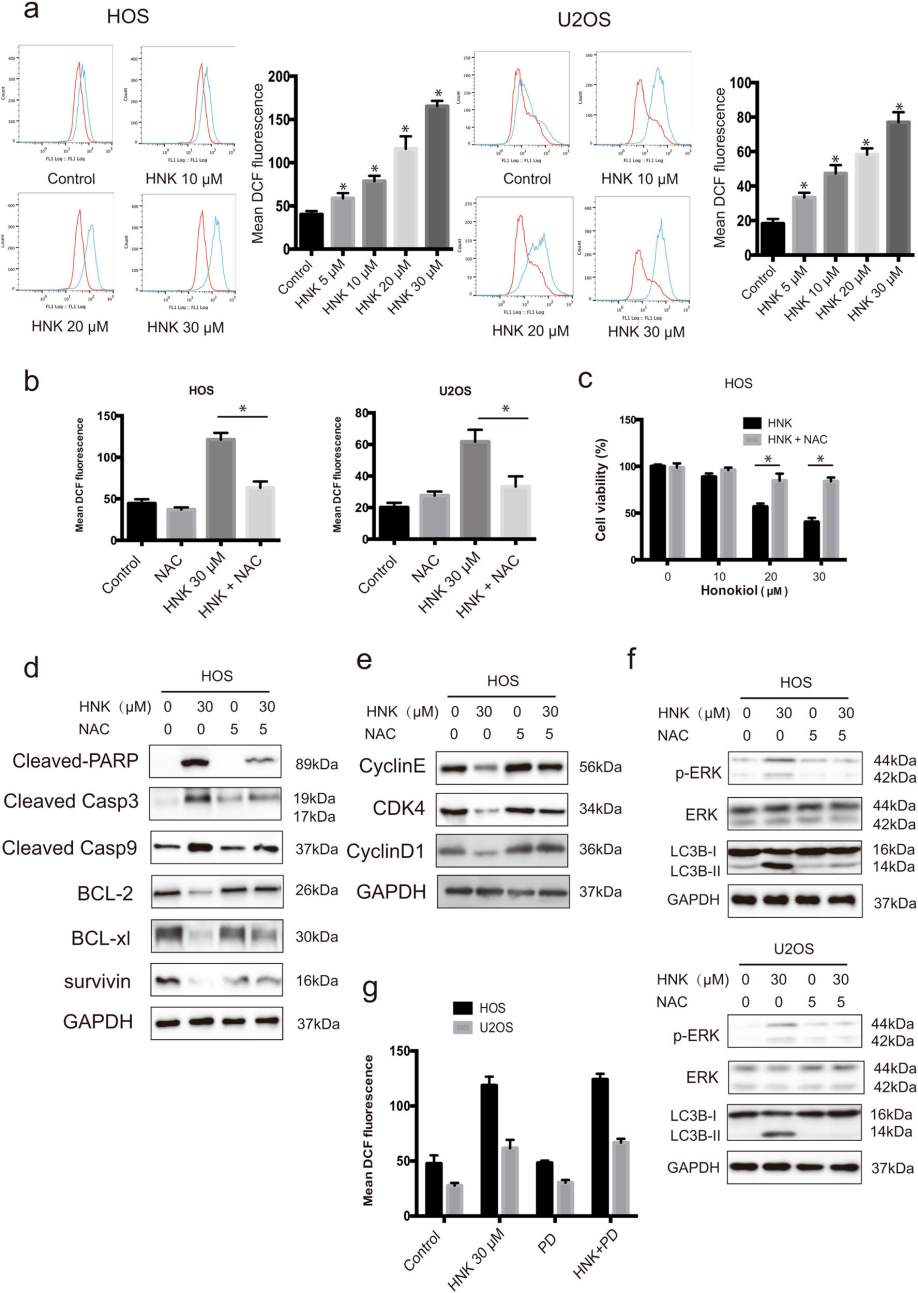
Roles of ROS in G0/G1 phase arrest, autophagy and apoptosis induced by HNK **a** Cells were treated with HNK for 24 h and then loaded with DCFH-DA for 30 min. The level of ROS was determined by flow cytometry. Representative images are presented. Quantitative analysis of ROS generation is shown in histograms. *P < 0.05 vs. control. **b** Cells were preincubated with NAC (5 mM) for 2 h, and then treated with HNK (30 μM) for 24 h. Level of ROS was determined by flow cytometry. **c** HOS cell viability was measured by MTT. **d** HOS cells were preincubated with NAC (5 mM) for 2 h, and then treated with HNK (30 μM) for 24 h. Levels of PARP, cleaved caspase-3, caspase-9, BCL-2, BCL-xl, and survivin were determined by western blot. **e** The expressions of HOS cell cycle-regulated proteins were measured by western blot. **f** Levels of phospho-ERK, *ERK*, and LC3B-II were determined by western blot. **g** HOS and U2OS cells were preincubated with PD98059 (40 μM) for 2 h, and then treated with HNK (30 μM) for 24 h. Level of ROS was determined by flow cytometry. Semi-quantification of western blot bands is presented in Figure S2c, f

For the reason that ROS can be induced by ER stress and is closely related to the regulation of apoptosis and cell cycle arrest, and also promote the sustained ERK activation, we detected the ROS level. As shown in Fig. 5a, ROS generation was initiated by 5 μM HNK and reached to peak by 20, 30 μM HNK at 24 h. A ROS scavenger was used to confirm the role of ROS in HNK-induced cell death. Figure 5b shows that NAC blocked the ROS accumulation induced by HNK. MTT assay showed that NAC rescued the cell death (Fig. 5c). Different from the effect of ERK inhibitor, NAC largely abolished the apoptosis rate and the diminishment of MMP (Fig. 4c, d). Besides, western blot analysis demonstrated that NAC completely inhibited HNK-induced activation of apoptosis-related proteins (Fig. 5d). NAC also had a hard inhibitory effect on HNK-induced G0/G1 phase arrest by reversing the key molecules of G0/G1 cell cycle regulator markers (Fig. 5e). Furthermore, NAC strongly blocked ERK phosphorylation while the ERK inhibitor did not affect ROS generation (Fig. 5f, g). All these results indicate that ROS is the proximal event of ERK and most likely acts as an initiator in HNK-induced apoptosis and G0/G1 phase arrest.

### Autophagy is mediated by ERK activation and ROS generation

To determine the importance of ERK activation and ROS generation in HNK-induced autophagy, we analyzed the level of LC3B-II in the presence of PD or NAC. As shown in Figs. 4e and 5f, both PD and NAC significantly suppressed the increased expression of LC3B-II induced by HNK. These results indicate that autophagy triggered by HNK is dependent on ERK activation and ROS generation.

### Activation of Atg7 contributes to HNK-induced autophagy

**Fig. 6:**
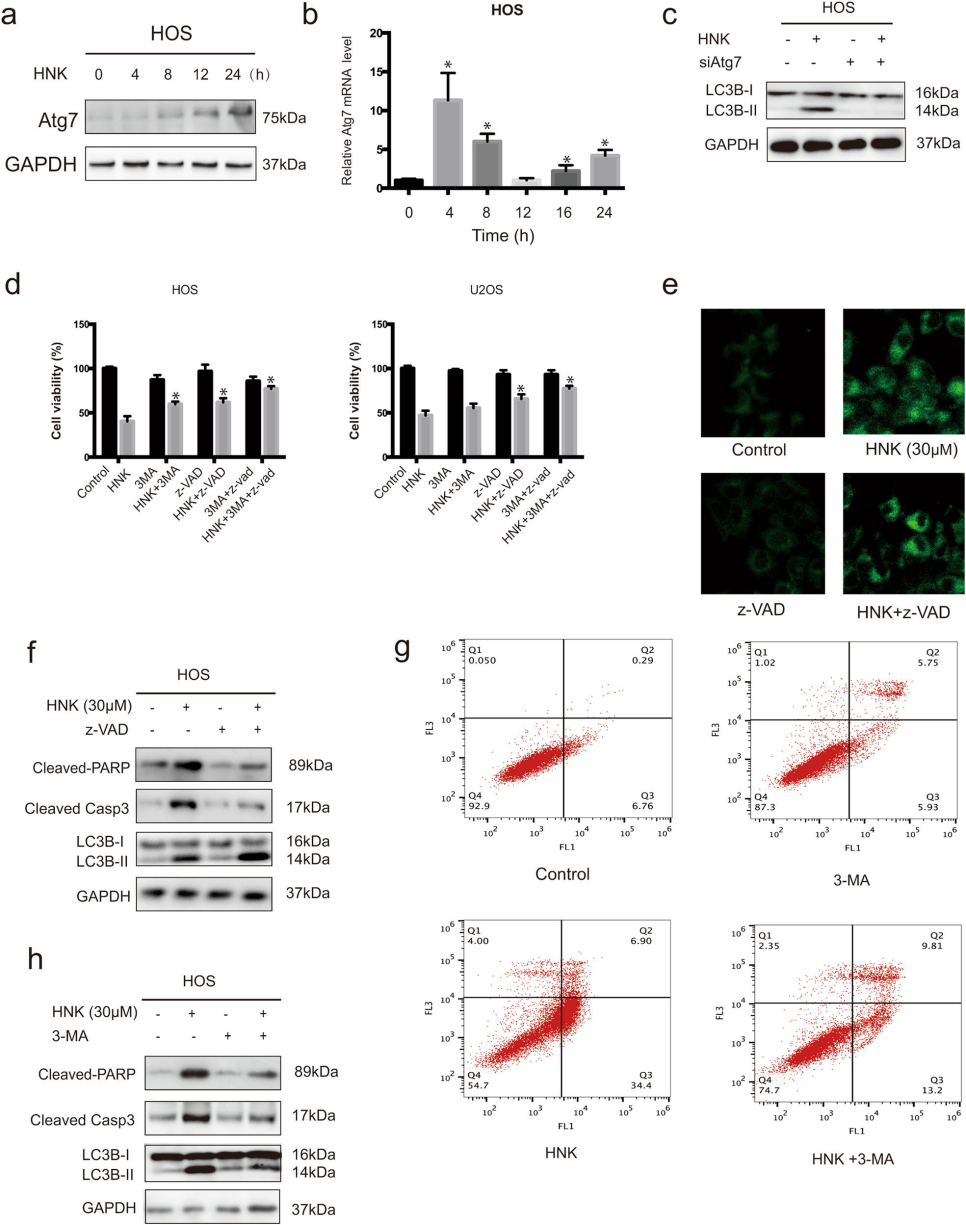
Roles of Atg7 in autophagy and the interplay between apoptosis and autophagy **a** Western blot of Atg7 in HOS cells treated with 30 μM honokiol for the indicated times. **b** Real-time PCR analysis of the expression of Atg7 in HOS cells. **c** Western blotting showed the repression of LC3B levels in HOS cells after siRNA-Atg7 treatment. **d** HOS and U2OS cells were preincubated with 3-MA (2.5 mM) or z-VAD-fmk (20 μM) for 2 h, and then treated with HNK (30 μM) for 24 h. Cell viability was measured by MTT. *P < 0.05. **e** The level of MDC in HOS cells are presented. **f** HOS cells were preincubated with z-VAD-fmk (20 μM) for 2 h, and then treated with HNK (30 μM) for 24 h. Levels of LC3B, cleaved PARP and caspase-3 were assessed by western blot. *P < 0.05 vs. control. **g** HOS cells were preincubated with 3-MA (2.5 mM) for 2 h, and then treated with HNK (30 μM) for 24 h. Cells were stained with annexin V-FITC/PI and analyzed by flow cytometry. **h** HOS cells were preincubated with 3-MA (2.5 mM) for 2 h, and then treated with HNK (30 μM) for 24 h. Levels of LC3B, cleaved PARP and caspase-3 were assessed by western blot. *P < 0.05 vs. control. Semi-quantification of western blot bands is presented in Figure S3a–d

Atg7 is reported to be a core regulator of autophagy, which is required for membrane trafficking and turnover in neuronal axons. Atg7 deficiency leads to multiple cellular abnormalities^42,43^. Therefore, we examined whether HNK treatment increased Atg7 protein levels in HOS cells. As shown in Fig. 6a, treatment with HNK enhanced the protein expression level of Atg7 in a time-dependent manner. Results from real-time PCR analysis indicated that Atg7 mRNA levels increased dramatically (about 10-fold) after a 4 h HNK treatment, and then decreased to the non-treated level after 12 h (Fig. 6b), when the protein level of Atg7 was increased instead (Fig. 6a). It is a common view that the mRNA changes several hours earlier than the protein changes. The regulation of Atg7 after HNK treatment corresponded with this point, which suggests that HNK transcriptionally regulates the expression of Atg7. However, the silencing of Atg7 with siRNA reduced HNK-induced LC3B-II protein levels, indicating that Atg7 is involved in HNK-induced autophagy in osteosarcoma cells (Fig. 6c). These results indicate that HNK-induced autophagy in HOS cells is dependent on the upregulation of Atg7 expression.

### Inhibition of apoptosis enhances autophagy while suppression of autophagy diminishes apoptosis

Overwhelming evidence has elucidated the complex relationship between apoptosis and autophagy^44^. To clarify the interplay between autophagy and apoptosis, cell viability in the presence of 3-MA, the autophagy inhibitor, was first assessed. We also analyzed cell viability in response to the combination of z-VAD-fmk and 3-MA to confirm the coactivation of these two cell death forms. As shown in Fig. 6d, 3-MA moderately diminished HNK-induced cell death. Interestingly, combination of z-VAD-fmk and 3-MA potently abolished the cell death. These data reveal that autophagy induced by HNK serves a pro-death function in osteosarcoma cells.

Then the effect of apoptosis inhibition on autophagy was determined. Figure 6e showed that z-VAD-fmk increased accumulation of MDC-stained AVs induced by HNK. As shown in Fig. 6f, although z-VAD-fmk inhibited HNK-induced activation of apoptosis-related proteins, z-VAD-fmk increased LC3B-II protein levels. Next, we examined the effect of autophagy inhibition on apoptosis. HNK-induced apoptosis was moderately blocked by 3-MA, despite slight apoptosis was observed in HOS cells treated with 3-MA alone (Fig. 6g). Figure 6h shows that 3-MA diminished cleavage of caspase-3 and PARP to a certain extent. These results suggest that inhibition of apoptosis enhances autophagy while autophagy might contribute to apoptosis.

### HNK inhibits growth of osteosarcoma in vivo

**Fig. 7:**
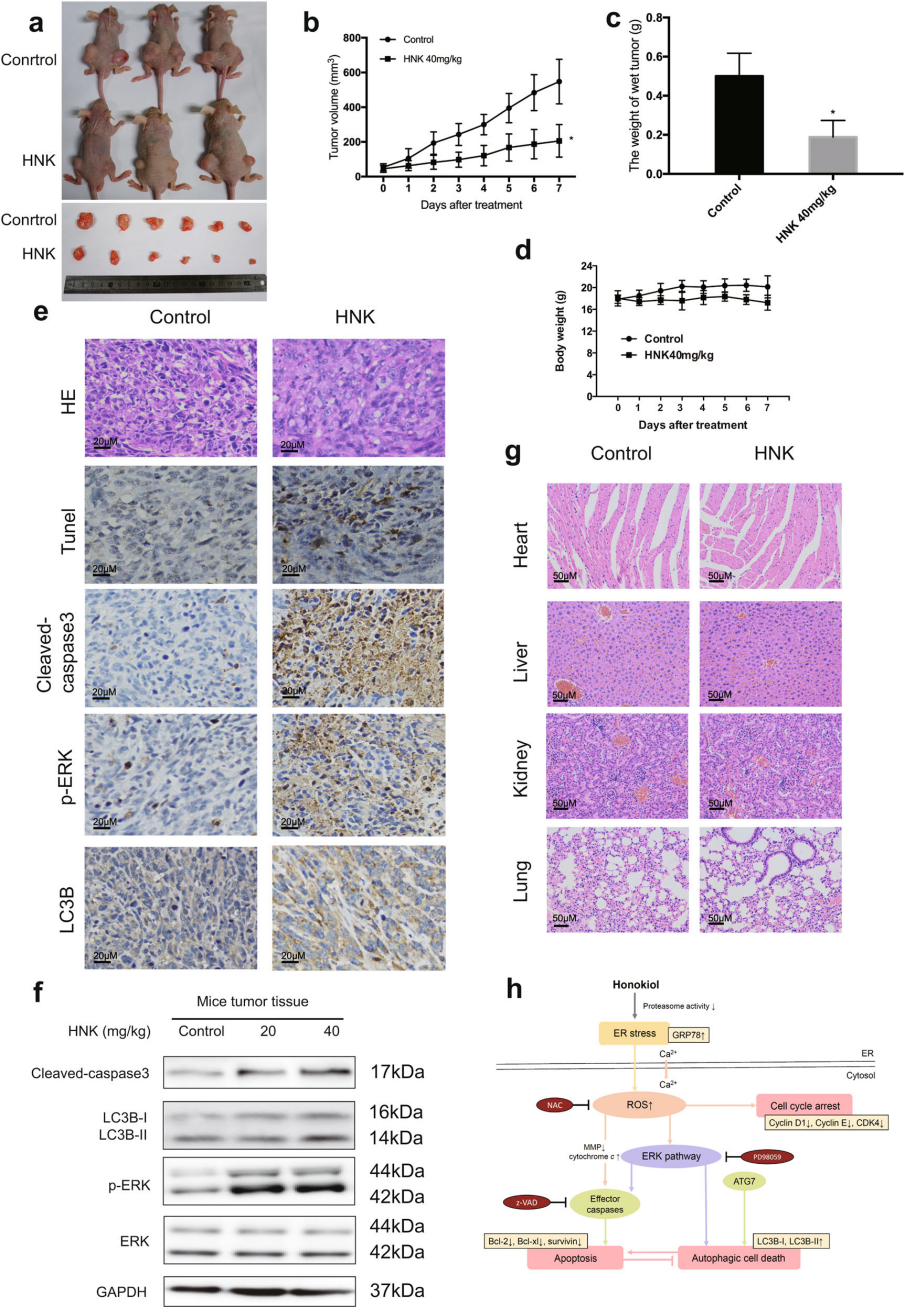
HNK inhibits OS xenograft growth in vivo HOS cells were injected into BALB/c-nu mice subcutaneously. One week after tumor inoculation, mice were randomly divided into two groups for treatment. Intraperitoneal administration of vehicle or HNK (40 mg/kg) every other day for seven times. **a**, **b** Tumor volume was measured every week. **c** HNK treatment resulted in significantly lower tumor weight than control group. **d** Body weights were measured every week. **e** The apoptotic status of tumor tissues was assessed by TUNEL assay. H&E staining was used to evaluate the histology. The expression levels of cleaved caspase-3, phospho-ERK, and* LC3B* were also examined by immunohistochemistry. Representative images were presented. **f** The levels of cleaved caspase-3, LC3B-I/II, phospho-ERK and total ERK in tumor xenograft tissues were measured by western blot. **g** No major organ-related toxicities were observed. H&E staining was used to evaluate the histology. **h** A model of the effects of honokiol on osteosarcoma cells. Semi-quantification of western blot bands is presented in Figure S3e

In vivo effect of HNK on osteosarcoma was determined via intraperitoneal administration in a tumor-transplanted mouse model. HNK at doses of 40 mg/kg resulted in significant decrease in tumor volume and weight, after 7 days of drug administration (Fig. 7a–c). However, no obvious decrease in body weight was observed in the experimental mice (Fig. 7d). Moreover, HNK-treated tumor tissues showed significant increase in TUNEL-positive cells, as well as the levels of cleaved caspase-3, ERK phosphorylation and LC3B (Fig. 7e). As shown in Fig. 7f, HNK treatment led to an increase in the levels of cleaved caspase-3, LC3B-II, and phospho-ERK. To investigate potential cytotoxic effects of HNK on normal tissues, non-tumor-bearing mice were intraperitoneally treated with HNK; hematoxylin and eosin (H&E) staining of organs collected at the end of the experiment revealed no major organ-related toxicities (Fig. 7g). These data show that HNK exhibited potent antitumor activity with low toxicity in vivo. Figure 7h showed that HNK induces apoptosis and autophagy via the ROS/ERK1/2 signaling pathway.

## Discussion

Owing to the new therapeutic developments, the prognosis of localized osteosarcoma has significantly improved. However, the long-term survival rate has stayed unchanged in the past several decades. Therefore, it is necessary to find novel therapeutics that can act effectively and efficiently through various anticancer mechanisms. In this study, we examined the anticancer effects of honokiol in osteosarcoma cells. We demonstrate that honokiol induces ROS-mediated autophagy and apoptosis in osteosarcoma cells. Furthermore, ERK activation via ROS production partially contributes to honokiol-induced cell death.

ROS, serving as important mediators, plays a critical role in regulating both cellular survival and death in response to different stimuli, such as starvation, chemotherapeutic agents, senescence, ionizing radiation, or protein misfolding^39,45–47^. ER stress can trigger ROS production through release of calcium. Although cancer cell proliferation can be stimulated by low doses of superoxide or hydrogen peroxide, irreversible damages in cancer cells could be induced by disproportionate cellular ROS levels through cell cycle arrest and apoptosis^39,48^. Moreover, enhanced mitochondrial oxidative stress results in caspases activation, cytochrome *c* release, and cell death^49^. Thus, based on the theory above, elevated intracellular ROS levels are used in many chemotherapeutics in order to induce cancer cell apoptosis^29^. In our study, honokiol treatment significantly increased intracellular ROS production, which has been suggested to be essential for both autophagy and apoptosis. Loss of MMP and increased PARP cleavage and caspase-3 activity, and decreased Bcl-2 expression were demonstrated. Besides, honokiol-induced cell death was completely reversed by ROS scavenger NAC. These data suggest the critical role of ROS in honokiol-induced anticancer effects.

MAPKs such as ERK and JNK, whose mechanism are multiple and complicated, are the downstream effects of ROS in autophagy induction^50,51^. However, in our study, honokiol treatment has no effect on JNK level (data not shown). As a member of the mitogen-activated protein kinase (MAPK) family, the ERK signaling pathway has been found playing an important role in various aspects of cell biological functions including proliferation, differentiation, migration, and death^52^. The ERK signaling pathway is able to be activated responding to various extracellular stimuli, including growth factors, mitogens, and cytokines, as well as immediate extracellular stresses, such as chemotherapy or radiation^53–55^. It is reported that the Ras/Raf/ERK signaling pathway has been regulated by ROS to modulate downstream AP-1 binding gene expression^56^. Generally, the ERK pathway activated by K-ras and growth factors has a significant role in cell proliferation in cancer^57^. However, some reports show that ROS-dependent ERK activation stirs up cancer cell cycle arrest and apoptosis. Prolonged ERK activation for apoptosis in various immortalized or transformed cells is required by anticancer chemotherapies such as etoposide and cisplatin^58^. Consistent with in vitro results, in a xenograft model ERK pathway mediated by ROS inhibited pancreatic tumor growth^59^. These growing evidences suggest that ERK activation also contributes to cell death. Consistently, in our present study, we found that treatment with 30 μM honokiol for 24 h induced ERK activation, and honokiol-induced autophagic cell death in osteosarcoma cells was at least partially dependent on ERK activity.

Autophagy, considered as a conserved catabolic process, disassembles dysfunctional and unnecessary cellular components under various stresses such as viral infection. The double membrane autophagosomes formation is morphological character of autophagy^60^, among which Atg5 and Atg7 were most significant. In this research, we found that protein levels of both Atg5 and Atg7 were increased after honokiol treatment. Our results indicate that honokiol-induced autophagy in osteosarcoma cells is dependent on the upregulation of Atg7. However, the detailed mechanism of the transcriptional upregulation of Atg7 will also require further investigation. Autophagy triggers either cell death or survival, making it a double-sided cellular process. In our results, z-VAD-fmk only caused a partial reduction in the HNK-induced cell death (Fig. 3a), and the autophagy inhibitor 3-MA (with or without z-VAD-fmk) partially rescued HNK-induced cell death (Fig. 6d), suggesting that autophagic pathway activation in HNK-treated cells leads to autophagic cell death. In case of extensive damages, another programmed cell death pathway (autophagic cell death) is promoted by autophagy^60^. Interestingly, honokiol-induced autophagy was inhibited by NAC co-treatment (Fig. 5f). Some researches indicate that several anticancer agents including resveratrol^61^ or cucurbitacin^62^ activate autophagy through ROS production and that the molecular mechanisms by which ROS induce autophagy. One of the mechanisms is that ROS oxidize Atg4 resulting in the enhanced autophagosome formation^63^. On the other hand, the interaction of Beclin-1 and Bcl-2 was disrupted by apogossypolone, which is accompanied by the increased ROS-mediated autophagy^27^. Therefore, honokiol might also increase the oxidation of Atg4 or interrupt the interaction of Beclin-1 and Bcl-2, thereby inducing autophagy activation. Further study will be needed to clarify this issue.

In conclusion, our results show that honokiol is a promising chemotherapeutic agent with a variety of anticancer effects. Honokiol treatment induced growth inhibition of osteosarcoma cancer cells in vitro and in vivo. Besides, honokiol treatment decreased the S and G2/M populations, increased the number of cells at G0/G1, and honokiol-induced apoptosis in osteosarcoma cells through the mitochondria dysfunction leading to activate caspase-9 and involves a caspase-3-mediated mechanism. Honokiol also induced ER stress, GRP78 activation, Ca^2+^ release. Moreover, the production of intracellular ROS, activation of the MAP kinase ERK, and upregulation of Atg7 were essential to the induction of honokiol-induced autophagy in osteosarcoma cells. These findings suggest that honokiol may be a potential clinical candidate for the treatment of osteosarcoma.

## Materials and methods

### Cell culture

HOS (ATCC: CRL-1543) and U2OS (HTB-96TM, ATCC) human osteosarcoma cells were obtained from the American Type Culture Collection (ATCC). The cells were cultured in Eagle’s minimum essential medium (MEM) (Gibco BRL, Grand Island, NY) supplemented with 10% (v/v) FBS and 1% (v/v) antibiotics (100 U/mL penicillin, 100 μg/mL streptomycin). The cells were maintained in an incubator set to 37 °C with 5% CO_2_.

### Reagents and antibodies

Honokiol was purchased from Wako (Osaka, Japan). 3-(4,5-dimethylthiazol-2-yl)−2, 5-Diphenyltetrazolium bromide (MTT), NAC, PD98059, and dimethyl sulfoxide (DMSO) were obtained from Sigma (St. Louis, MO, USA). MEM medium, fetal bovine serum (FBS), penicillin, streptomycin, and phosphate-buffered saline (PBS) were purchased from Gibco Life Technologies (Grand Island, NY, USA). Primary antibodies, including cleaved-poly (ADP-ribose) polymerase (PARP), Atg7, LC3B, p-ERK, ERK and GRP78, together with GAPDH antibodies and secondary antibodies, were purchased from Cell Signaling Technology, Inc. (Beverly, MA, USA). The broad-spectrum caspase inhibitor (z-VAD-fmk) was obtained from Millipore (Billerica, MA, USA). 3-MA was purchased from Selleckchem (Houston, TX, USA). Antibodies against caspase-3, caspase-9, Bcl-2, Bcl-xl, survivin, Cyclin D1, Cyclin E, and Cdk4 were purchased from Abcam.

### MTT assay

MTT assay was employed to examine the effects of HNK on the proliferation of osteosarcoma cells. Briefly, the cells were seeded in 96-well plates at 2 × 10^3^ cells per well in 100 μl medium. Then the cells in the wells were treated with various concentrations of HNK and cultured for 24, 48 or 72 h. At the end of culture, MTT solution (0.5 mg/mL in 20 μL PBS) was added to each well and incubated for 4 h at 37 °C. An enzyme-labeled instrument (Thermo) was used to measure the absorbance of each well at 570 nm. Data were calculated from three independent experiments, each performed in sextuplicate.

### Detection of acidic vesicular organelles

Formation of acidic vesicular organelles (AVOs), a morphological characteristic of autophagy, was detected by acridine orange (AO) staining. Cells were stained with 1 μg/ml acridine orange for 20 min and the samples were observed under a laser scanning confocal microscopy (excitation, 546 nm; emission, 575/640 nm).

### Visualization of autophagic vacuoles

The auto-fluorescent agent MDC was used as a specific autophagolysosome marker to analyze the autophagic process. Sarcoma cells were treated with different concentrations of HNK for 24 h. Autophagic vacuoles were labeled with MDC by incubating cells with 50 μM MDC in PBS at 37 °C for 20 min. After incubation, cells were washed three times with PBS and immediately analyzed by a laser scanning confocal microscopy (excitation, 390 nm; emission, 460 nm).

### Colony-formation assay

Cells were seeded in six-well plates at a density of 1000 cells per well. In the drug treatment group, the medium was changed with fresh medium containing HNK (10–30 μM) for about 14 days until the cells grew to visible colonies. Colonies were fixed with 4% paraformaldehyde and stained by crystal violet for 15 min at room temperature. The colonies that consisted of 450 cells were counted.

### Cell cycle analysis by flow cytometry

Cells were seeded in six-well plates with a density of 1 × 10^6^/ml and then treated with HNK at different concentrations for 24 h. After HNK treatment, the cells were harvested, washed with phosphate-buffered saline (PBS) and fixed with cold 75% ethyl alcohol at 4 °C overnight. The cells were then washed twice with PBS and incubated with RNase A for 30 min followed by staining with 500 μL propidium iodide for 30 min at room temperature. Cell cycle analysis was performed on the Accuri C6 (BD Biosciences, Mountain View, CA, USA).

### Mitochondrial membrane potential assay

The JC-1 Assay Kit (Beyotime, Beijing, China) was used to measure the alteration of mitochondrial membrane potential, according to the manufacturer’s instructions. Cells were seeded in six-well plates with a density of 5 × 10^5^/mL and then treated with HNK at concentrations ranging from 10–30 μM for 24 h. Then 100 μl of JC-1 staining solution was added into 1 mL of culture medium and incubated for 20 min at 37 °C in a CO_2_ incubator. The samples were analyzed by flow cytometry, and JC-1 aggregate was measured at the FL-2 channel and green fluorescent (both JC-1 monome) at the FL-1 channel (BD Biosciences).

### Apoptosis analysis by flow cytometry

Cells were seeded in six-well plates with a density of 1 × 10^6^/mL and then treated with HNK at concentrations ranging from 0 to 30 μM for 24 h. After HNK treatment, cells were harvested, washed twice with cold PBS and resuspended in the 1× binding buffer. Then, cells were incubated with FITC-conjugated Annexin V and PI for 15 min in the dark at room temperature, and the samples were analyzed using the flow cytometry in an hour (BD Biosciences).

### Transmission electron microscopy observation

Changes in cell ultra-structure caused by HNK were visualized using transmission electron microscopy (TEM). Apoptosis was assessed by observation of nuclear condensation and autophagy was evaluated by examining autophagosome formation. The treated cells were fixed with 2.5% glutaraldehyde and post-fixed with 1% osmium tetroxide. After being dehydrated in increasing concentrations of alcohol, the cell pellets were embedded in epon. Representative areas were chosen for ultrathin sectioning and examined on a transmission electron microscope at a magnification of ×5000.

### Measurement of ROS

Intracellular ROS production was detected by using the peroxide-sensitive fluorescent probe DCFH–DA. Cells were plated in six-well plates and treated with HNK at different concentrations in the absence or presence of 5 mM NAC. Cells were then incubated with DCFH–DA at a final concentration of 20 μM in MEM without FBS for 30 min at 37 °C and washed three times with MEM. The level of ROS was determined by flow cytometer (BD Biosciences; San Jose, CA, USA).

### Western blotting analysis

Cells were cultured in six-well plates at a density of 5 × 10^5^/mL per well and then treated with HNK (0–30 μM) for 24 h. Cells were washed with PBS, lysed in ice-cold RIPA containing a protease and a phosphatase inhibitor cocktail for 30 min on ice. Cell lysates were centrifuged at 12,000 × *g* for 15 min at 4 °C, and the supernatant was collected. Protein concentrations were quantified using the BSA Protein Assay according to the manufacturer’s instruction. Equal amounts (30 μg) of total protein were separated by SDS-PAGE (8–12%) at 100 V for 1.5 h and transferred to 0.45-μm PVDF membrane at 100 V for 1 h. After blocking with 5% non-fat milk in TBST buffer for 1 h at room temperature, the membranes were incubated with primary antibody at 4 °C overnight. The membranes were washed three times with TBST buffer and then incubated with peroxidase-conjugated secondary antibody for 1 h at room temperature. Specific antibody binding was detected by the Chemiluminescence Kit (Millipore, Plano, TX, USA).

### Xenograft

#### OS mouse model

Female BALB/c-nu mice (Shanghai Slac Laboratory Animal Co., Ltd., Shanghai, China) were purchased at 4 weeks of age and housed in a standard animal laboratory with free access to water and food. HOS cells were digested and washed by cold PBS for three times, and the final concentration was 1 × 10^7^/mL in cold PBS. A volume of 100 μL cell suspension was injected subcutaneously. When the tumors in the dorsal area were macroscopic, mice were randomly divided into three groups: control group and two HNK group (six mice in each group). Controls group received intraperitoneal injection of 100 μL 5% DMSO every other day, while HNK group was injected with 100 μL HNK (40 mg/kg, diluted with 5% DMSO). After seven times of drug administration, the mice were killed, and the tumors were removed, weighted, and fixed for use in immunohistochemical experiments. All the animal-related procedures were approved by the Animal Care and Use Committee of Sir Run Run Shaw Hospital.

### TUNEL assay

Apoptosis detection was identified using a TUNEL Assay Kit (Beyotime, Beijing, China) according to the manufacturer’s instructions. In brief, paraffin-embedded slides were deparaffinized with xylene and ethanol and rehydrated cell by proteinase K. After several washes with PBS, sections were incubated with TUNEL reaction mixture prepared freshly for 1 h at 37 °C in a moist chamber. Apoptotic cells on the slides were observed under an Olympus light microscope (Olympus, Tokyo, Japan) in randomly chosen fields.

### Histopathology and immunohistochemistry

Formalin-fixed tissue samples were embedded in paraffin and 4-μm sections were cut. Primary tumors, heart, liver, spleen, lung, and kidney sections were stained with H&E for routine histological examinations and morphometric analysis. For immunohistochemical staining, slides were deparaffinized in xylene and rehydrated with graded alcohol and incubated in 3% hydrogen peroxide to block the endogenous peroxidase activity. Antigen retrieval was performed by boiling the slides in 10 mM sodium citrate (pH 6.0) for 30 min. Then slides were blocked in 10% normal goat serum for 15 min, followed by incubation with p-ERK, and cleaved caspase-3 at 4 °C overnight in a moist chamber. On the next day, slides were washed in PBS and incubated with the second antibody for 1 h at room temperature. Immunoreactivity was detected using the Vectastain Elite DAB KIT (Vector Laboratories, Burlingame, CA, USA).

### Statistical analysis

Statistical analysis was performed using the SPSS version 18.0 software (IBM Corporation, Chicago, IL, USA). Student’s *t*-test, Fisher’s Exact test, and one-way ANOVA were used for calculating the significance between different groups. Statistical significance is indicated by *P* < 0.05. All data were expressed as mean ± s.d. of three independent experiments.

## Electronic supplementary material

Figure S1

Figure S2

Figure S3

Supplementary Information
