# Supplementary figures and images for: Honokiol induces apoptosis and autophagy via the ROS/ERK1/2 signaling pathway in human osteosarcoma cells in vitro and in vivo
Source: Cell Death Dis. 2018 Feb 6;9(2):157. doi: 10.1038/s41419-017-0166-5 (PMC5833587; doi:10.1038/s41419-017-0166-5)

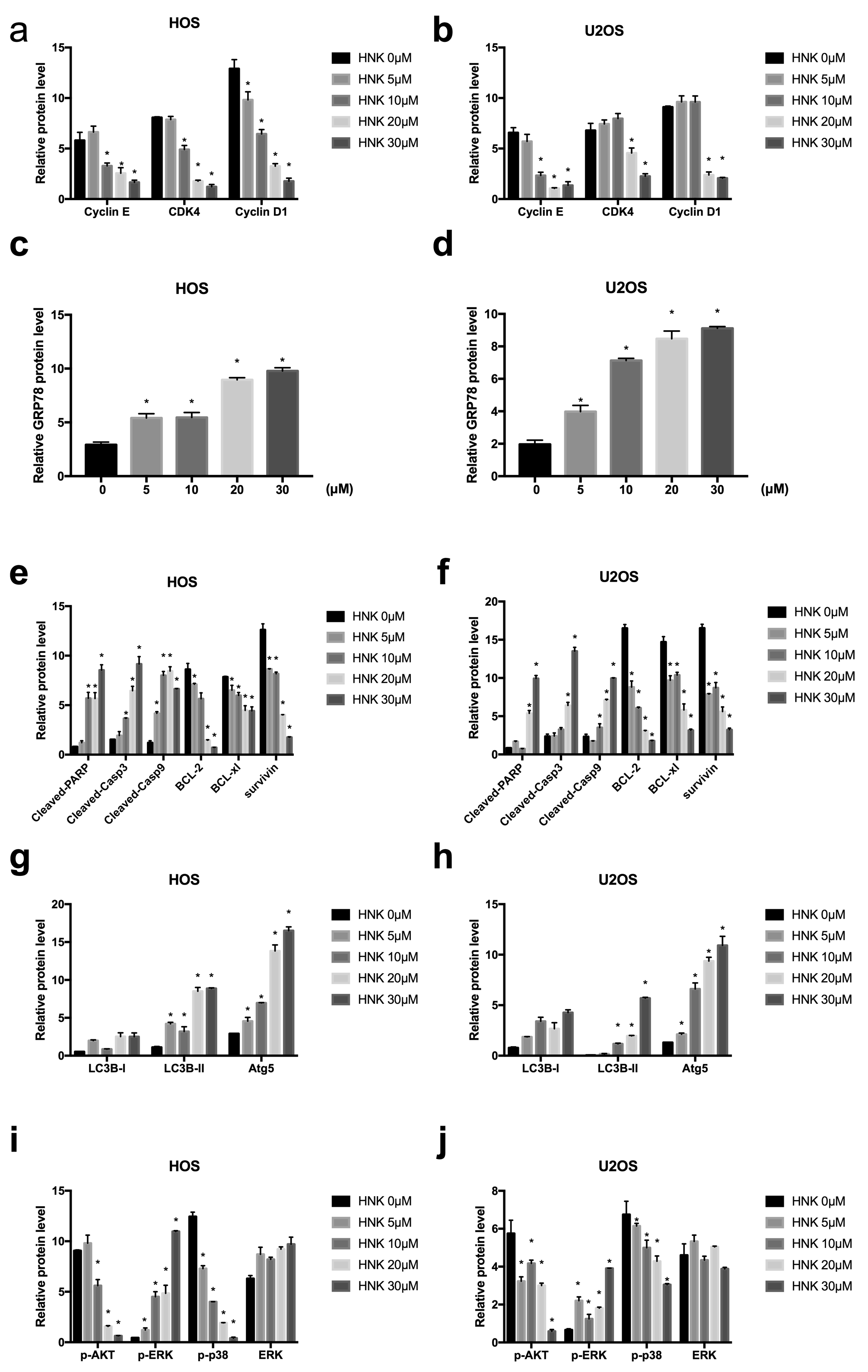

Supplement: Supplementary file 1 — Figure S1 [file 41419_2017_166_MOESM1_ESM.tif]

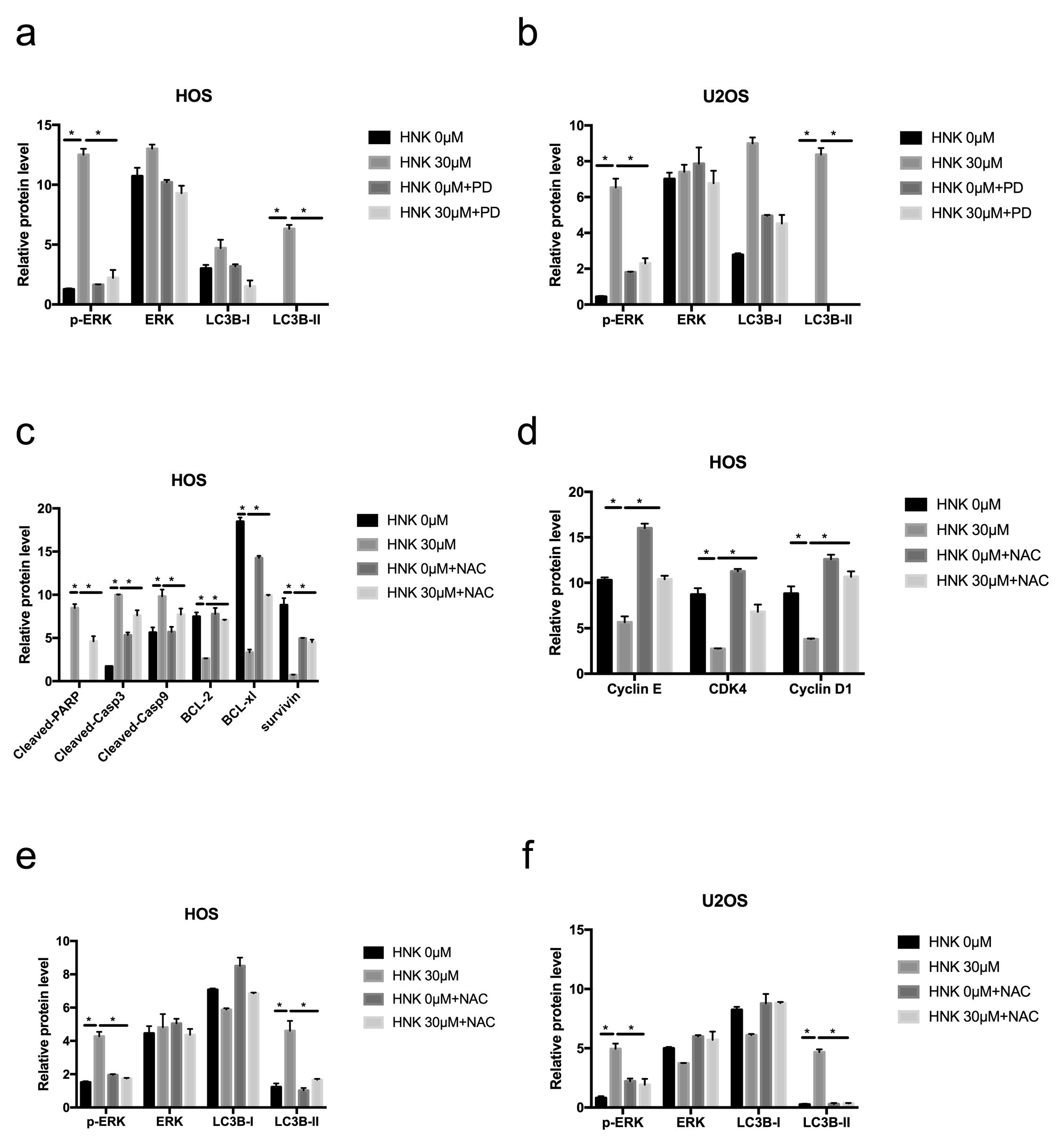

Supplement: Supplementary file 2 — Figure S2 [file 41419_2017_166_MOESM2_ESM.tif]

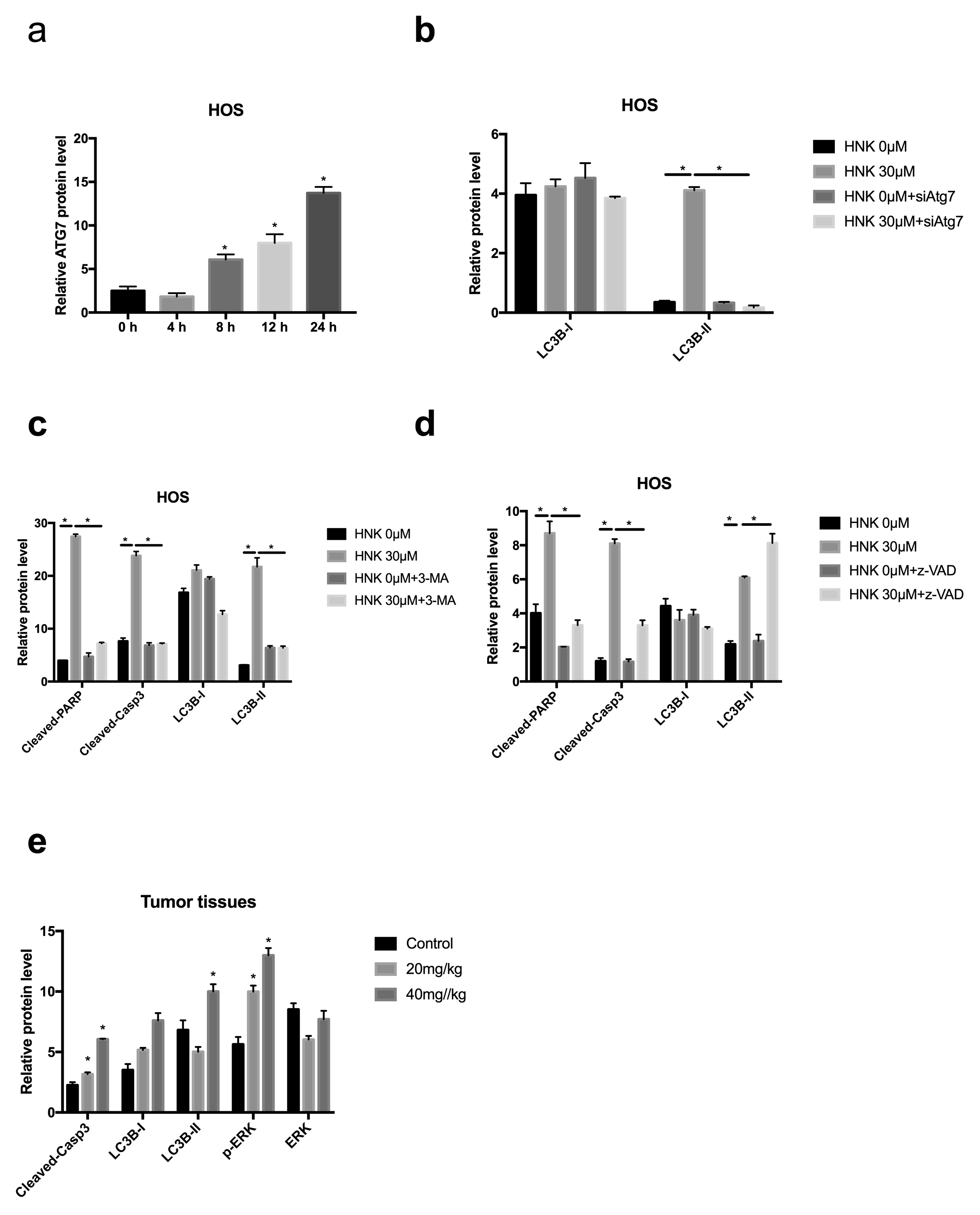

Supplement: Supplementary file 3 — Figure S3 [file 41419_2017_166_MOESM3_ESM.tif]
