# Supplementary material for: Honokiol induces apoptosis and autophagy via the ROS/ERK1/2 signaling pathway in human osteosarcoma cells in vitro and in vivo
Source: Cell Death Dis. 2018 Feb 6;9(2):157. doi: 10.1038/s41419-017-0166-5 (PMC5833587; doi:10.1038/s41419-017-0166-5)
Supplement: Supplementary file 4 — Supplementary Information [file 41419_2017_166_MOESM4_ESM.docx]

**SUPPLEMENTARY FIGURE LEGENDS**

**Figure S1. The relative protein levels of osteosarcoma cells treated with HNK**

HOS and U2OS treated with 0, 5, 10, 20 or 30 μM HNK for 24 h. (a-j) The relative protein levels of Cyclin E, CDK4, Cyclin D1, GRP78, Cleaved-PARP, Cleaved-Casp3, Cleaved-Casp9, BCL-2, BCL-xl, survivin, LC3B-I, LC3B-II, Atg5, p-AKT, p-ERK, p-p38 and ERK were semi-quantified using Image J. Corresponding western blot bands are shown in Figure 1-4.

**Figure S2. The relative protein levels of HNK-treated osteosarcoma cells preincubated with or without PD98059 and NAC**

(a-b) HOS and U2OS were preincubated with PD98059 (40 μM) for 2 h, and then treated with HNK (30 μM) for 24 h. Relative protein levels of phospho-ERK*,* ERK, LC3B-I and LC3B-II were determined by Image J. Corresponding bands are shown in Figure 4e. (c-f) HOS cells were preincubated with NAC (5 mM) for 2 h, and then treated with HNK (30 μM) for 24 h. Relative protein levels of cleaved PARP, caspase-3, -9, BCL-2, BCL-xl, survivin, cycle-regulated proteins, phospho-ERK, ERK, LC3B-I and LC3B-II were determined using Image J. Corresponding bands are shown in Figure 5d-f.

**Figure S3. The relative protein levels of osteosarcoma cells and tumor tissues**

(a) Relative protein levels of Atg7 in HOS cells treated with 30 μM honokiol for the indicated times. (b) Western blotting showed the repression of LC3B levels in HOS cells after siRNA-Atg7 treatment. (c) HOS cells were preincubated with 3-MA (2.5 mM) for 2 h, and then treated with HNK (30 μM) for 24 h. Levels of LC3B, cleaved PARP and caspase-3 were assessed by western blot. *P<0.05 versus control. *(d) HOS cells were preincubated with z-VAD-fmk (20 μM) for 2 h, and then treated with HNK (30 μM) for 24 h. Levels of LC3B, cleaved PARP and caspase-3 were assessed by western blot. *P<0.05 versus control. (e)*  *The protein levels of cleaved caspase-3, LC3B-I/II, phospho-ERK and total ERK in tumor xenograft tissues were measured by western blot. Corresponding bands are shown in Figure 6 and Figure 7f.*
